# Nutritional intra-amniotic therapy increases survival in a rabbit model of fetal growth restriction

**DOI:** 10.1371/journal.pone.0193240

**Published:** 2018-02-21

**Authors:** Hatice Gulcin Gumus, Miriam Illa, Laura Pla, Monica Zamora, Fatima Crispi, Eduard Gratacos

**Affiliations:** 1 Fetal i+D Fetal Medicine Research Center, BCNatal -Barcelona Center for Maternal-Fetal and Neonatal Medicine (Hospital Clinic and Hospital San Juan de Deu), Institut Clinic de Ginecologia, Obstetricia i Neonatalogia, Institut d'Investigacions Biomediques August Pi i Sunyer, University of Barcelona, Barcelona, Spain; 2 Centre for Biomedical Research on Rare Diseases (CIBER-ER), Barcelona, Spain; The University of Manchester, UNITED KINGDOM

## Abstract

**Objective:**

To evaluate the perinatal effects of a prenatal therapy based on intra-amniotic nutritional supplementation in a rabbit model of intrauterine growth restriction (IUGR).

**Methods:**

IUGR was surgically induced in pregnant rabbits at gestational day 25 by ligating 40–50% of uteroplacental vessels of each gestational sac. At the same time, modified-parenteral nutrition solution (containing glucose, amino acids and electrolytes) was injected into the amniotic sac of nearly half of the IUGR fetuses (IUGR-T group n = 106), whereas sham injections were performed in the rest of fetuses (IUGR group n = 118). A control group without IUGR induction but sham injection was also included (n = 115). Five days after the ligation procedure, a cesarean section was performed to evaluate fetal cardiac function, survival and birth weight.

**Results:**

Survival was significantly improved in the IUGR fetuses that were treated with intra-amniotic nutritional supplementation as compared to non-treated IUGR animals (survival rate: controls 71% vs. IUGR 44% p = 0.003 and IUGR-T 63% vs. IUGR 44% p = 0.02), whereas, birth weight (controls mean 43g ± SD 9 vs. IUGR 36g ± SD 9 vs. IUGR-T 35g ± SD 8, p = 0.001) and fetal cardiac function were similar among the IUGR groups.

**Conclusion:**

Intra-amniotic injection of a modified-parenteral nutrient solution appears to be a promising therapy for reducing mortality among IUGR. These results provide an opportunity to develop new intra-amniotic nutritional strategies to reach the fetus by bypassing the placental insufficiency.

## Introduction

Intrauterine growth restriction (IUGR) is generally defined as a significant reduction in fetal growth rate resulting in a birth weight in the lowest 10th percentile. It affects 7–10% of all pregnancies [1] and is considered a major contributor to perinatal morbidity and mortality, responsible for about 20–50% of perinatal deaths. It is also associated with worse short and long-term outcomes as increased prevalence of intrapartum distress, neonatal complications [2], suboptimal neurodevelopment [3,4] and cardiovascular disease [5,6]. Currently, there is no effective nutritional therapy to improve fetal growth or to ameliorate the adverse outcomes associated with IUGR [7–10]. Thus, the assessment of fetal well-being and timely delivery remain as the main management strategy, outweighing fetal injury/stillbirth versus the risks of iatrogenic preterm delivery.

Placental insufficiency is the most common cause of IUGR where the nutrient transport to the fetus is compromised [11]. To date, several studies in humans [10,12–15] and animals [16–18] testing diverse therapies administrated to the mother have failed to demonstrate a substantial improvement in fetal outcomes related to placental insufficiency. The lack of effectiveness of these therapies that are only aimed at the mothers could most probably be explained by a failure of nutrient transport between the mother and the fetus in the presence of the placental disease [19–22], therefore the administrated therapies cannot cross the placenta and reach the fetus. Direct nutrient supply to the fetus could theoretically overcome this problem by bypassing the placenta. However, previous studies that attempted to supply carbohydrates, growth factors or amino acid mixtures through trans-amniotic catheter insertion or direct fetal injections led to inconclusive results [23–30]. Moreover, most studies used a single nutrient approach with an invasive trans-amniotic placement of a catheter for several days. We hypothesized now that the administration of a complete nutrient composition (combining essential nutrients such as glucose, amino acids and electrolytes) in a single intra-amniotic injection could improve the outcomes of IUGR. Thus, we planned to administrate this complete nutrient composition therapy by intra-amniotic injections based on the fetus capacity of swallowing amniotic fluid, by which essential nutrients delivered intra-amniotically would reach the gastrointestinal tract and be absorbed [31,32], potentially compensating the nutrient deficiency caused by placental insufficiency.

In this study, we used a rabbit model of placental insufficiency to test the hypothesis that intra-amniotic nutrient delivery would improve the perinatal outcome of IUGR fetuses, by analyzing survival, birth weight and fetal cardiac remodeling.

## Material and methods

### Animals and experimental procedure

The study has been reported according to the ARRIVE guidelines[33] for reporting the in vivo experiments. Animal handling and all procedures were performed in accordance with applicable regulations and guidelines and with the approval of the Animal Experimental Ethics Committee of the University of Barcelona (Permit no: 250/15) All efforts were made to reduce both animal suffering and the number of animals used.

**Fig 1:**
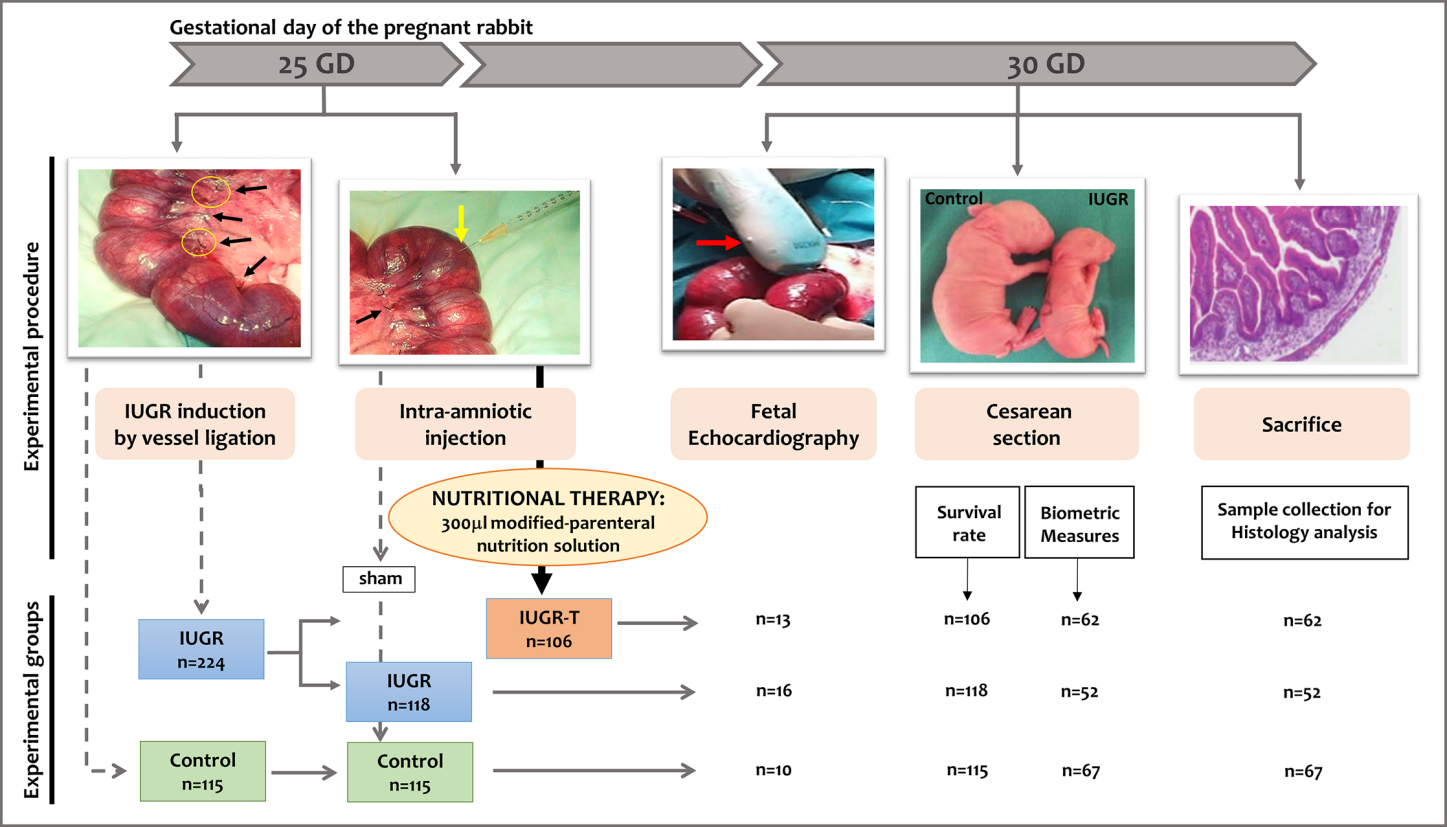
Experimental design. In pregnant rabbit at 25^th^ day of gestation, IUGR was surgically induced by uteroplacental vessel ligation and intra-amniotic injections were performed. Control fetuses did not undergo vessel ligature and they had sham injection. IUGR fetuses underwent uteroplacental vessel ligature and sham injection. and finally IUGR-T fetuses underwent uteroplacental vessel ligature and therapy administration which is intra-amniotic injection of 300 μl of modified-parenteral nutrient solution. At 30^th^ day of gestation, cesarean section was performed and uterine horns were exteriorized to perform fetal echocardiography in a subgroup of fetuses from each experimental group. The fetuses were then taken out for survival assessment and biometric measurements and then sacrificed for tissue sampling. Black arrows and yellow circles indicate ligated uteroplacental vessels of fetal sacs in the uterine horn, yellow arrow indicates intra-amniotic injection to the fetal sac and red arrow indicates the ultrasound transducer.

Thirty-eight time-mated 24 months old New Zealand White pregnant rabbits were provided by a certified breeder at 18^th^ day of gestation (full term is approximately 31 days). Dams were housed in separate cages on a reversed 12/12 h light cycle, with free access to water and standard chow. At 25^th^ day of gestation, IUGR was induced surgically by uteroplacental vessel ligation and intra-amniotic injections were performed. At 30^th^ day of gestation,an abdominal incision was made and the uterine horns were exteriorized to perform fetal echocardiography in a subgroup of fetuses. Subsequently, fetuses were delivered by cesarean section. Experimental design and timeline are shown in Fig 1 and the description of all the procedures are detailed in the following sections.

### Rabbit model of IUGR and therapy administration

On gestational day 25, an abdominal midline laparotomy was performed and both uterine horns were exteriorized under endovenous anesthesia of Ketamine (Ketolar® 50mg/ml, Pfizer, 10 mg/kg) and Xylazine (Rompun® 2%, Bayer, 3mg/kg). Gestational sacs of both horns were counted and numbered, and each fetus was identified according to the fetal position within the bicornuate uterus. Prior to surgery, each uterine horn was randomly allocated to a group (control, IUGR or IUGR-T) based on a computer generated randomization number sequence. As each dam has two uterine horns, in order to obtain three experimental groups (control, IUGR and UGR-T), horns of each dam was assigned to a paired combination of these groups, resulting in three combinations (control and IUGR, control and IUGR-T, or IUGR and IUGR-T). Based on the ligation and nutrient injection, the experimental groups were created: Control (no IUGR induction and sham injection, n = 115), IUGR (IUGR induction and sham injection, n = 118) and IUGR-T (IUGR induction and therapy administration, n = 106). IUGR was surgically induced by ligation of 40–50% of the uteroplacental vessels of the assigned gestational sacs [34]. In addition, 300 μl of modified-parenteral nutrition solution (see Table 1 for composition details: a complete mixed composition containing glucose, amino acids and electrolytes, but excluding lipids, based on previous evidence of respiratory insufficiency in fetuses who received trans-amniotic lipid emulsion [26]), was injected to the amniotic sac of the IUGR-T fetuses via a 25G needle (B.Braun Sterican®). The IUGR and control groups received needle puncture without administrating any substance to the amniotic sac (sham injection) (Fig 1). Administration of Buprenorphine (Buprex injectable, 0.3 mg/ml; Schering-Ploug, Madrid, Spain) was used as a post-operative medication: The dams received a single dosage of Buprenorphine (0.01–0.05mg/kg), administrated subcutaneously after the induction of IUGR and administrated orally diluted in the water during the first 48 hours after the operation (0.03ml /5kg/8h).

**Table 1:** Composition of the modified-parenteral nutrition solution (per 30 mL).

Glucose (g)	3.7
Amino acids(g)	1.1
L-Isoleucine (g/L)	8
L-Leucine (g/L)	13
L-Lysine Monoacetate (g/L)	12
L-Lysine (g/L)	8.5
L-Methionine (g/L)	3.1
L-Phenylalanine (g/L)	3.8
L-Threonine (g/L)	4.4
L-Tryptophan (g/L)	2.0
L-Valine (g/L)	9
L-Arginine (g/L)	7.5
L-Histidine (g/L)	4.8
Glycine (g/L)	4.2
L-Alanine (g/L)	9.3
L-Proline (g/L)	9.7
L-Serine (g/L)	7.7
Taurine (g/L)	0.4
N-acetyl-L-tyrosine (g/L)	5.2
L-Tyrosine (g/L)	4.2
N-Acetyl-L-Cysteine (g/L)	0.7
L-Cysteine (g/L)	0.5
L-Malic Acid (g/L)	2.6
Sodium (mEq)	0.6
Calcium (mEq)	0.7
Magnesium (mEq)	0.2
Chlorine (mEq)	0.3
Phosphor (mmol)	0.2
Acetate (mEq)	0.7
Carnitine (mg)	3.7
Heparin (UI)	15.3

The solution has an osmolarity of 1.085 mOsm/L

### Fetal echocardiography and cesarean section

At 30 days of pregnancy, an abdominal midline laparotomy was performed and uterine horns exteriorized under endovenous anesthesia of Ketamine (Ketolar® 50mg / ml, Pfizer) 10 mg/kg and Xylazine (Rompun® 2%, Bayer) 3mg/kg. After that, fetal echocardiography was performed in a subgroup of fetuses using vivid q ultrasound equipment (GE Healthcare, Little Chalfont Buckinghamshire, UK) with i12L-RS linear transducer, placed directly on each exteriorized gestational sac. Cardiac area, thoracic area, ventricular base-to-apex length and transverse diameters, and septal myocardial wall thickness were measured in end-diastole from a 2D image. Then, cardio-thoracic ratio was calculated by dividing cardiac area per thoracic area. Ventricular sphericity indexes were calculated as base-to-apex length divided by transverse diameter. Heart rate was also measured using Doppler applied on the left outflow tract.

Immediately after echocardiography, all live and stillborn fetuses were obtained by uterine horn incision and weighted. Dams were sacrificed by endovenous overdose of sodium pentobarbital (200 mg/kg), immediately after fetal extraction. All living newborns were sacrificed by immediate decapitation. Survival rate was determined by the ratio of live fetuses at the time of the cesarean section to all viable fetuses at the time of the ligature procedure. Intestine samples were collected after delivery for subsequent analysis.

### Sampling and analysis of fetal intestine

After sacrificing the fetuses, one-centimeter tissue sample was collected from the proximal small intestine and fixed with 4% paraformaldehyde in PBS for 24 h at 4°C. Fixed intestinal samples were embedded in paraffin to obtain 5μm sections to be stained with hematoxylin and eosin. Histology images were acquired using a microscope (Leica, Bannockburn, IL) and software (Leica Application Suite, version 3.4). Quantification of intestine diameter, villus height and muscular and sub-mucosal layer thickness was performed using Image J software (http://rsbweb.nih.gov/ij) in order to evaluate intestinal structure.

### Statistical analysis

The STATA14.0 package was used for statistical analyses. Qualitative variables were compared by Pearson’s Chi Square test. Normal distribution of quantitative variables was assessed by Shapiro-Wilk test. Normally-distributed variables were expressed as mean and standard deviation and analyzed by one-way ANOVA followed by a Bonferroni’s Multiple Comparison post hoc test. Non-normal distributed parameters were shown as median and interquartile range and compared by non-parametric Kruskal-Wallis. Statistical significance was declared at p<0.05.

## Results

### Intra-amniotic nutrient supplementation increases IUGR survival with no improvement in birth weight

A total of 339 fetuses were obtained (115 control, 106 IUGR and 108 IUGR-T fetuses, respectively) from 38 dams. Of these 339 fetuses, 201 were alive at the day 30 of cesarean section (82 controls, 52 IUGR and 67 IUGR-T). The mean litter size was 11.4 ± 2.3.

**Fig 2:**
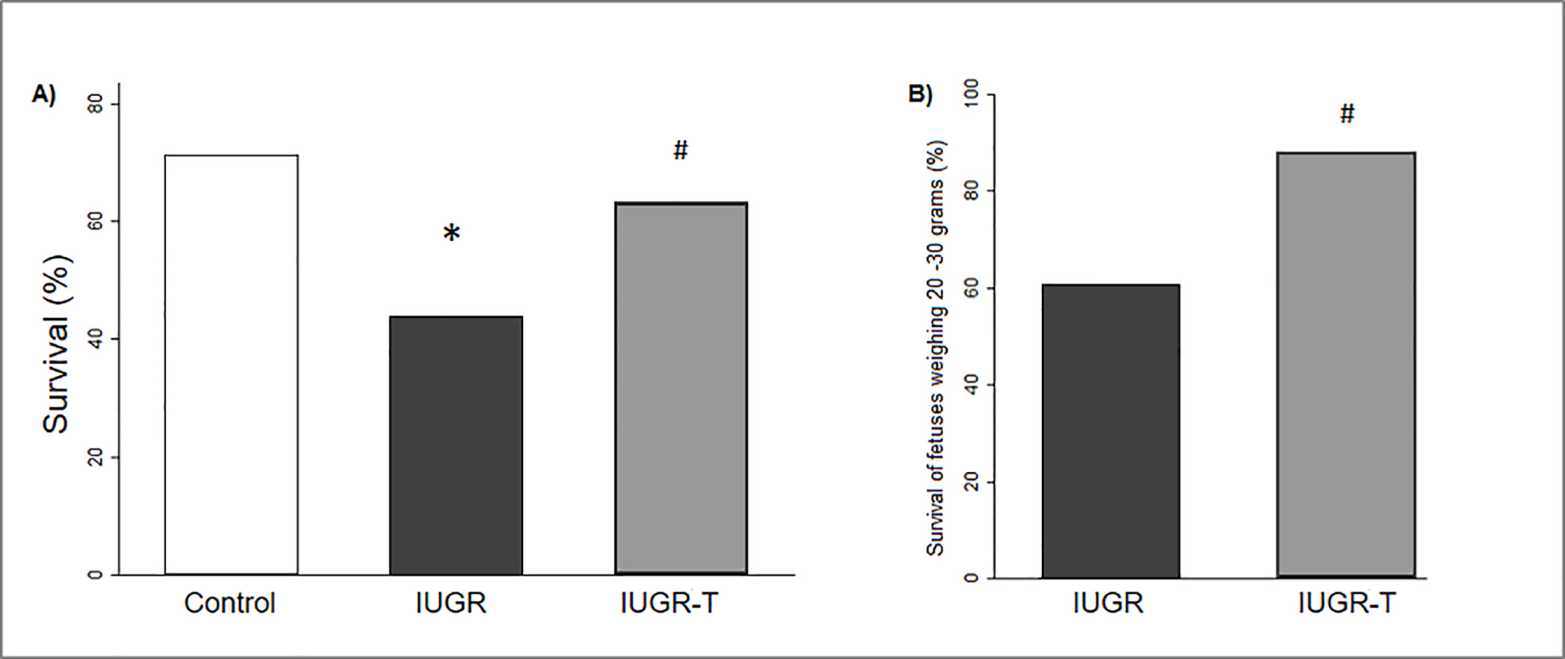
Survival rate. Survival rate among controls, intrauterine growth restricted cases (IUGR) or IUGR cases with therapy (IUGR-T) in the overall population (Control: 82 surviving out of 115; IUGR: 52 surviving out of 118; IUGR-T: 67 surviving out of 106) (A) and among newborns weighing 20-30g (IUGR: 14 surviving out of 23; IUGR-T: 22 surviving out of 25) (B). * p<0.05 as compared to controls; ^#^ p<0.05 as compared to IUGR.

**Fig 3:**
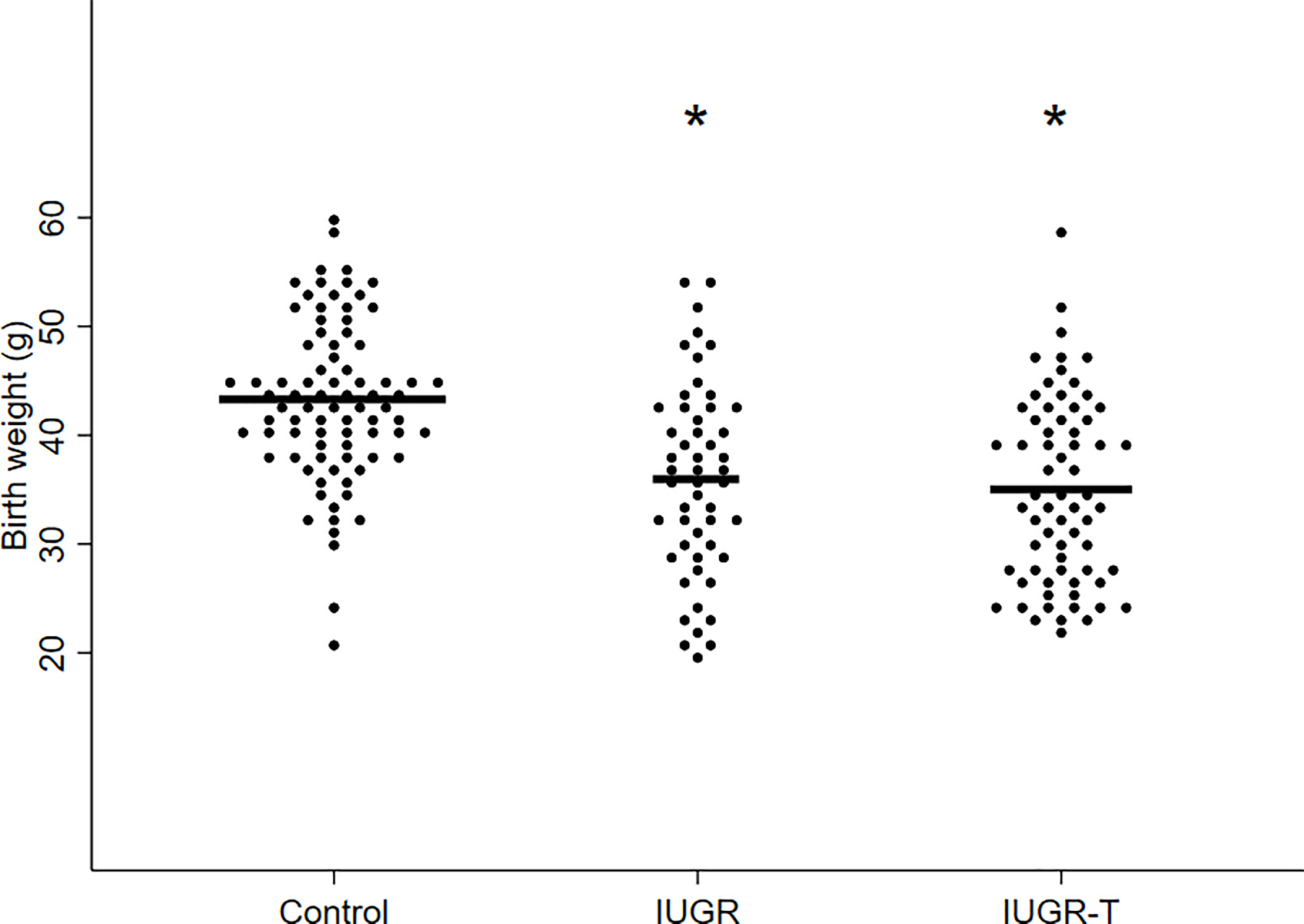
Birth weight. Scatter dot plot of birth weight among controls, intrauterine growth restricted cases (IUGR) or IUGR cases with therapy (IUGR-T). The horizontal line is the mean.* p<0.05 as compared to controls.

**Fig 4:**
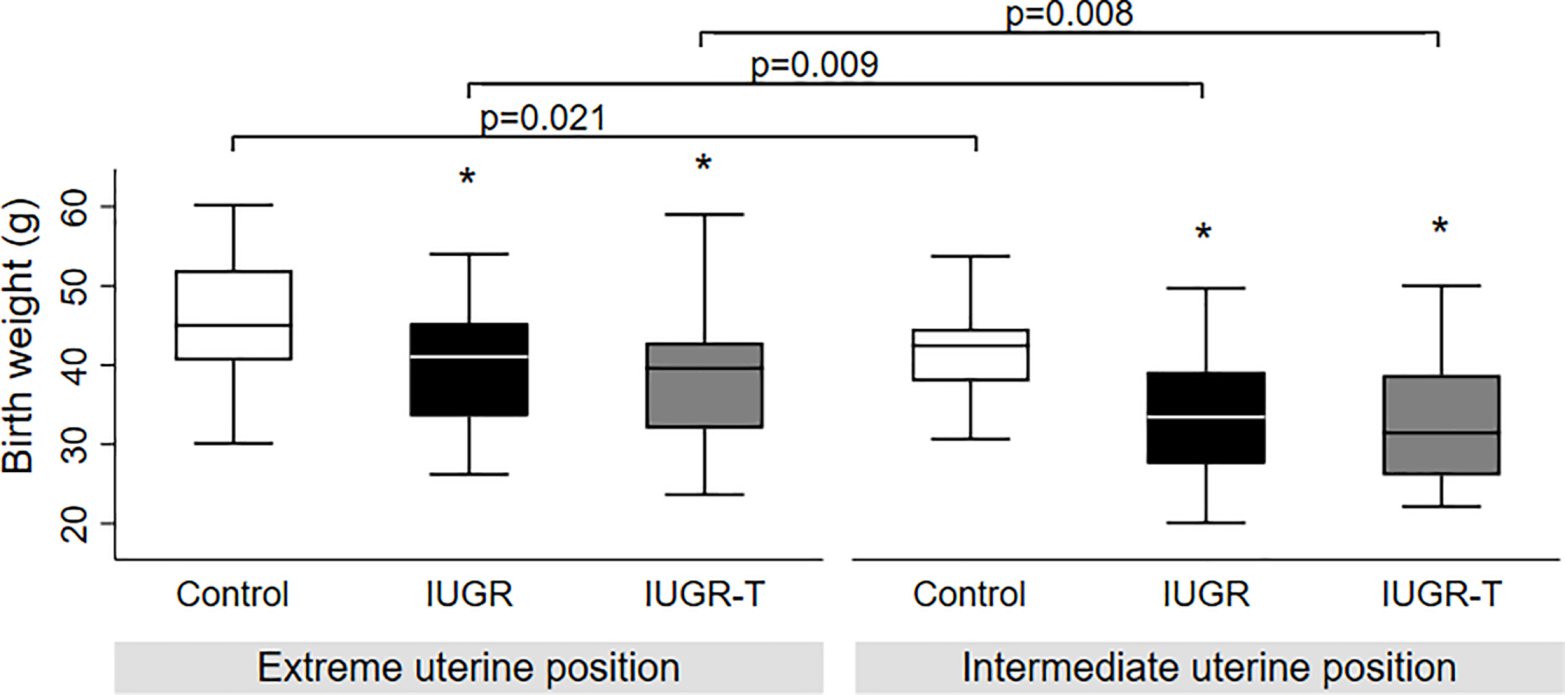
Birth weight in relation to uterine position. Birth weight among controls, intrauterine growth restricted cases without (IUGR) or with therapy (IUGR-T) in fetuses that are in extreme positions (ovarian and cervical ends) (Control n = 32; IUGR n = 20; IUGR-T n = 25) (A) and intermediate positions (Control n = 50; IUGR n = 32; IUGR-T n = 42) (B), * p<0.05.

**Fig 5:**
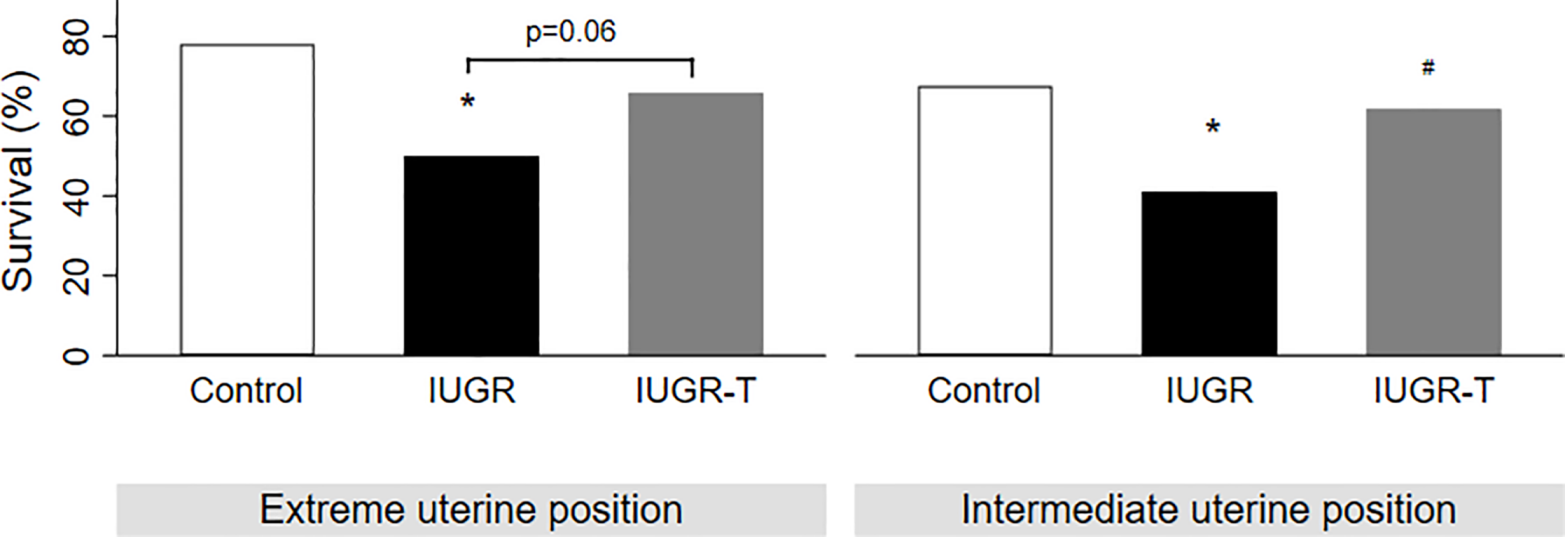
Survival rate in relation to uterine position. Survival rate among controls, intrauterine growth restricted cases without (IUGR) or with therapy (IUGR-T) in fetuses that are in extreme positions (Control: 32 surviving out of 41; IUGR 20 surviving out of 40; IUGR-T 25 surviving out of 38) and intermediate positions (Control: 50 surviving out of 74; IUGR: 32 surviving out of 78; IUGR-T: 42 surviving out of 68) positions. * p<0.05 as compared to controls; ^#^ p<0.05 as compared to IUGR.

Non-treated IUGR fetuses presented a significantly lower survival rate (IUGR 44% vs. control 71% p = 0.003) and lower birth weight as compared to controls (Figs 2A and 3). However, under therapy, IUGR-T fetuses showed a significantly higher rate of survival (IUGR-T 63% vs. IUGR 44%, p = 0.02) despite the birth weight was similar to non-treated IUGR (Figs 2A and 3). A further analysis associated with fetuses’ uterine position revealed that birth weight of control fetuses was significantly higher as compared to both IUGR and IUGR-T groups independently from the uterine position (Fig 4). As expected, fetuses in extreme positions (ovarian and cervical ends) had higher survival rate as compared to the fetuses in intermediate positions (control 78% versus 68%; IUGR 50% versus 41%; IUGR-T 66% versus 62%, Fig 5) as compared to the fetuses in the intermediate positions. This observation was detected in all the experimental groups. For both positions, control fetuses had significantly higher birth weight than IUGR and IUGR-T fetuses, while birth weight of the latter two groups did not differ from each other (Fig 4).

An analysis performed for a subgroup of fetuses that weighed less than 30 grams (which correspond to the 10^th^ centile of normally distributed weight at birth [3,6,34–36]) revealed that a significantly higher proportion of IUGR-T animals with that weight were alive compared to IUGR animals (Fig 2B).

### Intra-amniotic nutrient supplementation does not compensate for fetal cardiac adaptation

Fetal echocardiography revealed similar cardiac alterations in both IUGR and IUGR-T fetuses with larger hearts, thicker myocardial walls and a more spherical left ventricle as compared to controls (Table 2).

**Table 2:** Fetal echocardiographic results in the studied groups.

	Control (n = 10)	IUGR (n = 16)	IUGR-T (n = 13)
Cardio-thoracic area	0.35 ± 0.03	0.40 ± 0.06*	0.39 ± 0.06*
Myocardial wall thickness^†^	0.17 ± 1.01	0.21 ± 0.05*	0.20 ± 0.07*
LV sphericity index	1.43 ± 0.05	1.32 ± 0.05*	1.22 ± 0.06*
RV sphericity index	1.22 ± 0.17	1.26 ± 0.21	1.19 ± 0.09

Values are mean ± standard deviation.

*p<0.05 as compared to the control group.

†Myocardial wall thickness normalized by cardiac area. LV, left ventricular; RV, right ventricular.

### Intra-amniotic nutrient supplementation ameliorates IUGR intestine structural changes

**Fig 6:**
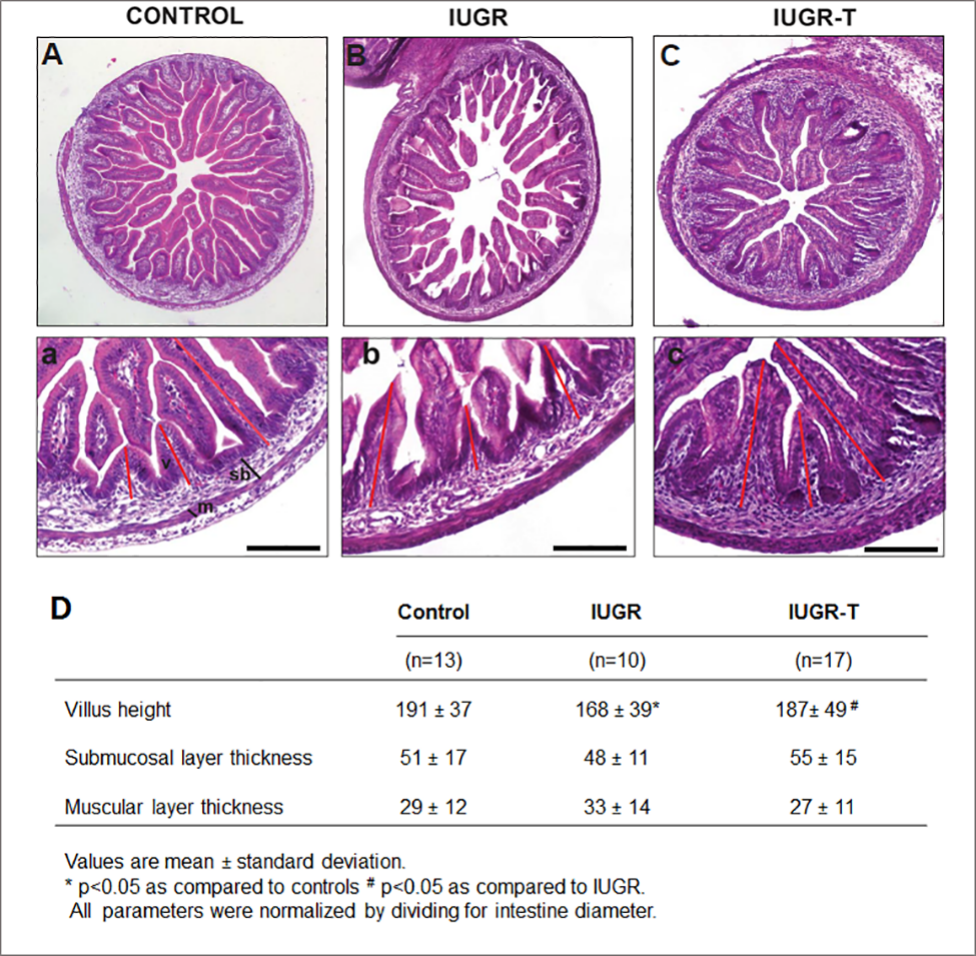
Histological analysis of the small intestine. Hematoxylin and eosin stained sections from controls (A), intrauterine growth restricted cases (IUGR, B) or IUGR cases with therapy (IUGR-T, C), illustrating similar villus height in controls and IUGR-T, with shorter height in IUGR. (a,b,c) 200 x magnification of stained sections, scale bars correspond to 100μm. sb denotes sub-mucosa and m mucosa. (D) Intestinal morphometric measurements of controls, intrauterine growth restricted cases without (IUGR) or with therapy (IUGR-T).

Regardless of the absence of any positive change in fetal cardiac functions and birth weight among IUGR-T treated fetuses, the notable improvement in the survival rate in this group versus IUGR fetuses may suggest that the nutritional supplementation administrated was able to reach circulation in IUGR fetuses, very likely through swallowing and intestinal absorption. Actually, performed histological analyses of the small intestine (Fig 6) provide some evidence for that hypothesis; revealing shorter villus height and a less organized structure of the absorbent surface in IUGR than IUGR-T fetuses, which appears to be partially ameliorated in IUGR-T intestines (Fig 6).

## Discussion

Our results support intra-amniotic injection of nutrients as a promising therapy for reducing mortality among IUGR, regardless of no apparent effects on birth weight. These results open opportunities for intra-amniotic nutritional strategies to reach the fetus bypassing the placenta.

The striking finding of our study is the improvement of survival rate in IUGR fetuses receiving intra-amniotic nutritional supplementation. Our results demonstrate that intra-amniotic injection of a modified-parenteral nutrition notably reduces mortality in an animal model of placental insufficiency. In contrast, intra-amniotic nutrition was not able to ameliorate birth weight or fetal cardiac adaptation. Several studies in the early 90s also attempted to supply nutrients in the amniotic cavity with dissimilar results. Mulvihill et al. demonstrated a positive effect on fetal growth with similar mortality by 5-days intra-amniotic continuous infusion of bovine amniotic fluid or dextrose plus amino acids in rabbits [27,28]. In contrast, Flake et al. could not demonstrate any improvement in birth weight by 6-days continuous amniotic infusion of dextrose, dextrose-amino acid mixture or lipids in a ‘natural runting’ IUGR rabbit model [26]. Actually, the infusion of lipid emulsion resulted in chronic lipid aspiration and further growth retardation. Phillips et al. used 4-days continuous intra-amniotic infusion of radioactive glucose and proline to demonstrate fetal nutrients absorption but failed to show changes in survival and birth weight [30]. Buchmiller et al. showed unchanged body weight and mortality after 4-days intra-amniotic infusion of galactose [37]. Finally, in the present study a combination of carbohydrates, amino acids and electrolytes were administrated by a single amniotic injection in a rabbit model of uteroplacental vessel ligation showing improvement of survival despìte no improvement in birth weight. Overall, the contradictory results from different studies could be explained by differences in therapy duration (single administration vs. 4–6 days of continuous infusion) and timing, type of nutrients administered (including or not including electrolytes), IUGR models (naturally vs. uteroplacental ligation) and sample size. Nutrient administration by a single injection might associate less mortality than a more invasive procedure such as a catheter insertion required for continuous infusion during several days. While intra-amniotic lipids seem deleterious, carbohydrates and amino acids appear essential for fetal development and growth. In addition, electrolytes such as potassium, calcium, magnesium could also be essential for fetal survival by regulating nutrient uptake [38]. The use of large sample size in a severe IUGR model with high perinatal mortality enabled us to demonstrate an improvement in survival rate among IUGR-T fetuses. We speculate that the specific mixture of glucose, amino acids and electrolytes (without lipids) administrated by a single amniotic injection in the present study is enough to improve the fetal nutritional status subsequently increasing survival. Our data also suggest that nutritional status seems to be more critical than hypoxia for survival. A possible explanation for the lack of birth weight improvement in IUGR-T fetuses could be that survival of mainly the more severely restricted animals (fetuses with birth weight between 20 and 30 grams that would otherwise have died) pulled down the mean birth weight. Another potential explanation is that single administration of nutrients could only partially counteract the effect of placental insufficiency. Placental insufficiency is usually associated with a complex pathophysiologic adaptation leading to nutrient and oxygen restriction to the fetus, but also increased placental resistance inducing pressure overload to the fetal heart (that has to pump against a more resistant placenta). Most likely, intra-amniotic injection of modified-parenteral nutrition permits to ameliorate the critical fetal nutritional deficiency, but not the fetal hypoxia or pressure overload (that would explain the maintained low birth weight and fetal cardiac remodeling).

Uterine horn position seems to be a relevant factor for birthweight in rabbit model. Bautista and colleagues [39] demonstrated that animals closer to the extremities of the uterine horn had higher weight and survival compared to the animals in intermediate position. In correlation with the results reported by Bautista et al., we have also found that fetuses in the extreme positions had significantly higher birth weight than the fetuses in the intermediate position in all groups (Fig 4). Moreover, the birth weight difference of the fetuses between subgroups was significant which is consistent with our results of the whole population (control fetuses had significantly higher birth weight than IUGR and IUGR-T fetuses, while the birth weight was similar in IUGR and IUGR-T), independently of the position. We have also observed a non-significant trend for higher survival rate in extreme positions (Fig 5) which is also consistent with previously reported data [39]. Taken together, the position analysis indicates that the therapy is effective to counteract IUGR, independent of the uterine position.

The present study also showed that IUGR induction by uteroplacental vessels ligation had a negative impact on the gut structure that seems to be ameliorated by intra-amniotic injection of nutrients. Similarly, previous studies exhibited improved small intestine growth in IUGR animals by esophageal infusions of nutrients to fetal rabbit and fetal sheep [28,29], demonstrating the nutritive value of fetal swallowing in fetal intestine. In addition, previous data suggest that intra-amniotic infusion of nutrients swallowed by the fetus are transported through the gastrointestinal tract, absorbed and concreted into fetal tissues [30], suggesting an active transport of nutrients in the fetal small intestine. Taken together, our findings correlate with these studies and provide additional support for the hypothesis that intra-amniotic infusion of nutrients has a trophic effect in fetal small intestine which might provide an additional explanation for the increase in survival with IUGR-T fetuses in our study.

We acknowledge gender issues as one potential limitation for our study as sex of the animals could not be determined at the time of birth, therefore it was not possible to analyze differences in survival rate and birth weight by gender. As it was stated in the study of Tarrade et al.[40], sexual dimorphism can be often observed in rabbits in the HFD model. Further studies are needed to assess the difference between male and female birth weight and survival rate in the nutritional intra-amniotic therapy model. We also acknowledge limited information on the weights of placenta of newborn rabbits, therefore we could not calculate the fetal-placental weight ratio (F:P). Future studies are warranted to examine the impact of intra-amniotic therapies in placental development.

In conclusion, our study demonstrates that intra-amniotic nutrient supplementation increases survival rates of IUGR fetuses remarkably, particularly among those more severe IUGR animals. The use of a mixed nutritional solution containing essential carbohydrates, amino acids and electrolytes seems as an appropriate approach for reducing the mortality in IUGR. The findings from our study could be considered as a potential advance to fetal intervention of IUGR. This would raise the possibility of therapeutic strategies to improve survival particularly in those more severely restricted fetuses. Future studies are warranted to evaluate different fetal nutritional supplementation in IUGR outcomes in order to find the optimal mode, dose and timing of administration and nutritional composition.

## References

[1] M Kady S, Gardosi J. Perinatal mortality and fetal growth restriction. Best Pract Res Clin Obstet Gynaecol. 2004;18: 397–410. doi: 10.1016/j.bpobgyn.2004.02.009 15183135 10.1016/j.bpobgyn.2004.02.009

[2] Bernstein IM, Horbar JD, Badger GJ, Golan A, Ohisson A. Intrauterine Growth Restriction in Very Low Birth Weight Newborns: Neonatal Outcome. Am J Obstet Gynecol. 1997;176: 176. 10.1016/s0002-9378(00)70513-8 10649179

[3] Illa M, Eixarch E, Batalle D, Arbat-Plana A, Muñoz-Moreno E, Figueras F, et al Long-Term Functional Outcomes and Correlation with Regional Brain Connectivity by MRI Diffusion Tractography Metrics in a Near-Term Rabbit Model of Intrauterine Growth Restriction. PLoS One. 2013;8 doi: 10.1371/journal.pone.0076453 24143189 10.1371/journal.pone.0076453 PMC3797044

[4] Eixarch E, Batalle D, Illa M, Muñoz-Moreno E, Arbat-Plana A, Amat-Roldan I, et al Neonatal neurobehavior and diffusion MRI changes in brain reorganization due to intrauterine growth restriction in a rabbit model. PLoS One. 2012;7: 1–12. doi: 10.1371/journal.pone.0031497 22347486 10.1371/journal.pone.0031497 PMC3275591

[5] Crispi F, Bijnens B, Figueras F, Bartrons J, Eixarch E, Le Noble F, et al Fetal growth restriction results in remodeled and less efficient hearts in children. Circulation. 2010;121: 2427–2436. doi: 10.1161/CIRCULATIONAHA.110.937995 20497977 10.1161/CIRCULATIONAHA.110.937995

[6] Torre I, González-Tendero A, García-Cañadilla P, Crispi F, García-GarciÁa F, Bijnens B, et al Permanent cardiac sarcomere changes in a rabbit model of intrauterine growth restriction. PLoS One. 2014;9: 1–8. doi: 10.1371/journal.pone.0113067 25402351 10.1371/journal.pone.0113067 PMC4234642

[7] Gulmezoglu AM, Hofmeyr GJ. Plasma volume expansion for suspected impaired fetal growth. Cochrane database Syst Rev. ENGLAND; 2000; CD000167 doi: 10.1002/14651858.CD000167 10796166 10.1002/14651858.CD000167 PMC7045282

[8] Gulmezoglu AM, Hofmeyr GJ. Betamimetics for suspected impaired fetal growth. Cochrane database Syst Rev. England; 2001; CD000036 doi: 10.1002/14651858.CD000036 11687064 10.1002/14651858.CD000036 PMC7052749

[9] Laurin J, Persson PH. The effect of bedrest in hospital on fetal outcome in pregnancies complicated by intra-uterine growth retardation. Acta Obstet Gynecol Scand. SWEDEN; 1987;66: 407–411. 3321864 10.3109/00016348709022043

[10] Say L, Gulmezoglu AM, Hofmeyr GJ. Maternal nutrient supplementation for suspected impaired fetal growth. Cochrane database Syst Rev. England; 2003; CD000148 doi: 10.1002/14651858.CD000148 12535390 10.1002/14651858.CD000148 PMC7045469

[11] Wu G, Bazer FW, Cudd TA, Meininger CJ, Spencer TE. Maternal Nutrition and Fetal Development. J Nutr. 2004; 2169–2172. 15333699 10.1093/jn/134.9.2169

[12] Makrides M, Crowther C a., Gibson R a., Gibson RS, Skeaff CM. Efficacy and tolerability of low-dose iron supplements during pregnancy: A randomized controlled trial. Am J Clin Nutr. 2003;78: 145–153. 12816784 10.1093/ajcn/78.1.145

[13] Simmer K, Thompson RP. Maternal zinc and intrauterine growth retardation. Clin Sci (Lond). ENGLAND; 1985;68: 395–399. 3971668 10.1042/cs0680395

[14] Merialdi M, Carroli G, Villar J, Abalos E, Gülmezoglu a M, Kulier R, et al Nutritional interventions during pregnancy for the prevention or treatment of impaired fetal growth: an overview of randomized controlled trials. J Nutr. 2003;133: 1626S–1631S. Available: http://www.ncbi.nlm.nih.gov/pubmed/12730476 12730476 10.1093/jn/133.5.1626S

[15] Liberato SC, Singh G, Mulholland K. Effects of protein energy supplementation during pregnancy on fetal growth: a review of the literature focusing on contextual factors. Food Nutr Res. 2013;57. 10.3402/fnr.v57i0.20499 PMC3827488 24235913

[16] Somm E, Larvaron P, van de Looij Y, Toulotte A, Chatagner A, Faure M, et al Protective effects of maternal nutritional supplementation with lactoferrin on growth and brain metabolism. Pediatr Res. 2013;75: 51–61. doi: 10.1038/pr.2013.199 24213624 10.1038/pr.2013.199

[17] Lin G, Wang X, Wu G, Feng C, Zhou H, Li D, et al Improving amino acid nutrition to prevent intrauterine growth restriction in mammals. Amino Acids. 2014;46: 1605–23. doi: 10.1007/s00726-014-1725-z 24658999 10.1007/s00726-014-1725-z

[18] Lassala A, Bazer FW, Cudd TA, Datta S, Keisler DH, Satterfield MC, et al Parenteral Administration of L -Arginine Prevents Fetal Growth Restriction in Undernourished Ewes. J Nutr. 2010;140: 1242–1248. doi: 10.3945/jn.110.125658 20505020 10.3945/jn.110.125658 PMC2884328

[19] Marconi AM, Paolini CL. Nutrient Transport Across the Intrauterine Growth-Restricted Placenta. Semin Perinatol. 2008;32: 178–181. doi: 10.1053/j.semperi.2008.02.007 18482618 10.1053/j.semperi.2008.02.007

[20] Cetin I, Alvino G, Radaelli T, Pardi G. Fetal nutrition: a review. Acta Paediatr Suppl. 2005;94: 7–13. doi: 10.1080/08035320510043709 16214758 10.1111/j.1651-2227.2005.tb02147.x

[21] Wallace JM, Bourke D a, Aitken RP, Milne JS, Hay WW. Placental glucose transport in growth-restricted pregnancies induced by overnourishing adolescent sheep. J Physiol. 2003;547: 85–94. doi: 10.1113/jphysiol.2002.023333 12562948 10.1113/jphysiol.2002.023333 PMC2342623

[22] Regnault TRH, Friedman JE, Wilkening RB, Anthony R V, Hay WW. Fetoplacental transport and utilization of amino acids in IUGR—a review. Placenta. 2005;26 Suppl A: S52–62. doi: 10.1016/j.placenta.2005.01.003 15837069 10.1016/j.placenta.2005.01.003

[23] Harrison MR, Villa RL. Trans-amniotic fetal feeding. I. Development of an animal model: continuous amniotic infusion in rabbits. J Pediatr Surg. 1982;17: 376–80. Available: http://www.ncbi.nlm.nih.gov/pubmed/6811718 6811718 10.1016/s0022-3468(82)80493-4

[24] Fowden a L, Hughes P, Comline RS. The effects of insulin on the growth rate of the sheep fetus during late gestation. Q J Exp Physiol. 1989;74: 703–14. Available: http://www.ncbi.nlm.nih.gov/pubmed/2687925 2687925 10.1113/expphysiol.1989.sp003322

[25] Skarsgard ED, Amii L a, Dimmitt R a, Sakamoto G, Brindle ME, Moss RL. Fetal therapy with rhIGF-1 in a rabbit model of intrauterine growth retardation. J Surg Res. 2001;99: 142–6. doi: 10.1006/jsre.2001.6120 11421616 10.1006/jsre.2001.6120

[26] Flake AW, Villa-Troyer RL, Adzick NS, Harrison MR. Transamniotic Fetal Feeding III. The Effect of Nutrient Infusion on Fetal Growth Retardation. J Pediatr Surg. 1986;21: 481–484. 3088253 10.1016/s0022-3468(86)80216-0

[27] Mulvihill SJ, Albert A, Synn A, Fonkalsrud EW. In utero supplemental fetal feeding in an animal model: effects on fetal growth and development. Surgery. United States; 1985;98: 500–505. 4035570

[28] Mulvihill SJ, Stone MM, Fonkalsrud EW, Debas HT. Trophic effect of amniotic fluid on fetal gastrointestinal development. J Surg Res. 1986;40: 291–296. doi: 10.1016/0022-4804(86)90189-7 3702386 10.1016/0022-4804(86)90189-7

[29] Trahair J, Sangild P. Fetal organ growth in response to oesophageal infusion of amniotic fluid, colostrum, milk or gastrin-releasing peptide: a study in fetal sheep. Reprod Fertil Dev. 2000;12: 87–95. 11194563 10.1071/rd00024

[30] Phillips JD, Fonkalsrud EW, Mirzayan A, Kim CS, Kieu A, Zeng H, et al Uptake and distribution of continuously infused intraamniotic nutrients in fetal rabbits. J Pediatr Surg. 1991;26: 374–380. doi: 10.1016/0022-3468(91)90982-Y 2056396 10.1016/0022-3468(91)90982-y

[31] Pritchard J. Fetal Swallowing and Amniotic Fluid Volume. Obstet Gynecol. 1966; 606–610. 5332288

[32] Orlic D. Fetal Rat Intestinal Absorption of Horseradish Peroxidase from Swallowed Amniotic Fluid. J Cell Biol. 1973;56: 106–119. 4118449 10.1083/jcb.56.1.106 PMC2108829

[33] Kilkenny C, Browne WJ, Cuthill IC, Emerson M, Altman DG. Improving bioscience research reporting: The arrive guidelines for reporting animal research. Animals. 2013;4: 35–44. doi: 10.3390/ani4010035 10.1016/j.joca.2012.02.010 22424462

[34] Eixarch E, Figueras F, Hernández-Andrade E, Crispi F, Nadal A, Torre I, et al An experimental model of fetal growth restriction based on selective ligature of uteroplacental vessels in the pregnant rabbit. Fetal Diagn Ther. 2009;26: 203–11. doi: 10.1159/000264063 19955698 10.1159/000264063

[35] Eixarch E, Hernandez-Andrade E, Crispi F, Illa M, Torre I, Figueras F, et al Impact on fetal mortality and cardiovascular Doppler of selective ligature of uteroplacental vessels compared with undernutrition in a rabbit model of intrauterine growth restriction. Placenta. Elsevier Ltd; 2011;32: 304–309. doi: 10.1016/j.placenta.2011.01.014 21334065 10.1016/j.placenta.2011.01.014

[36] Illa M, Brito V, Pla L, Eixarch E, Arbat-Plana A, Batallé D, et al Early Environmental Enrichment Enhances Abnormal Brain Connectivity in a Rabbit Model of Intrauterine Growth Restriction. Fetal Diagn Ther. 2017; 1–10. doi: 10.1159/000481171 29020672 10.1159/000481171

[37] Buchmiller TL, Fonkalsrud EW, Kim CS, Chopourian HL, Shaw KS, Lam MM, et al Upregulation of Nutrient Transport in Fetal Rabbit Intestine by Transamniotic Substrate Administration ‘. 1992;447: 443–447. 10.1016/0022-4804(92)90309-n 1619911

[38] Ashworth CJ, Antipatis C. Review Micronutrient programming of development throughout gestation. Reproduction. 2001;122: 527–535. 11570959

[39] Bautista A, Rödel HG, Monclús R, Juárez-romero M, Cruz-sánchez E, Martínez-gómez M, et al Physiology & Behavior Intrauterine position as a predictor of postnatal growth and survival in the rabbit. Physiol Behav. Elsevier Inc.; 2015;138: 101–106. doi: 10.1016/j.physbeh.2014.10.028 25447330 10.1016/j.physbeh.2014.10.028

[40] Tarrade A, Rousseau-Ralliard D, Aubrière MC, Peynot N, Dahirel M, Bertrand-Michel J, et al Sexual Dimorphism of the Feto-Placental Phenotype in Response to a High Fat and Control Maternal Diets in a Rabbit Model. 2013;8 doi: 10.1371/journal.pone.0083458 24386205 10.1371/journal.pone.0083458 PMC3873307

